# Identification and characterization of miRNAs during early pregnancy in domestic sheep

**DOI:** 10.1111/age.12992

**Published:** 2020-08-13

**Authors:** Kisun Pokharel, Jaana Peippo, Meng‐Hua Li, Juha Kantanen

**Affiliations:** ^1^ Natural Resources Institute of Finland (Luke) Jokioinen Finland; ^2^ College of Animal Science and Technology, China Agricultural University Beijing China

**Keywords:** *corpus luteum*, endometrium, Finnsheep, preimplantation, Texel

## Abstract

MicroRNA resources in sheep are limited compared with those in other domesticated mammalian species. By sequencing small RNAs of sheep *corpus luteum* and endometrium, we have generated the largest amount of miRNA‐seq data and compiled the most comprehensive list thus far of miRNAs (*n* = 599) in sheep. Additionally, we observed a highly conserved maternally imprinted cluster of miRNAs on chromosome 18 homologous to that found on chromosome 14 in human and several other eutherian mammals.

A total of 18 ewes representing Finnsheep, Texel and their F_1_ crosses were included in the study. All of the procedures and experimental operations involving animals were performed according to the Finnish laws and EU directives regarding animal experimentation (Decision no. ESAVI/3853/04.10.03/2011 by The National Animal Experiment Board in Finland). After 2–3 weeks of pregnancy, the ewes were slaughtered to collect the tissue biopsies of *corpus luteum* (CL) and endometrium for inclusion in this study. Procedures for RNA extraction, sample quality control measurements and sequence library preparation have been described previously (Hu *et al.,* 2015; Pokharel *et al.,* 2020). High‐quality libraries of miRNA were sequenced with an Illumina HiSeq 2000 system using single‐end (1 × 50) sequencing strategy.

The raw sequence data were initially screened to obtain an overview of the data quality, including the 31 presence or absence of adapters, using FastQC version 0.11.6. (https://github.com/sandrews/FastQC). Next, the Illumina adapters and low‐quality bases were removed using Trim galore version 0.5.0 (https://github.com/FelixKrueger/TrimGalore). In addition, reads that were too short (having fewer than 18 bases) after trimming were discarded. To reduce downstream computational time, high‐quality reads were collapsed using seqcluster version 1.2.4a7 (Pantano *et al.,* 2011). The FASTQ output from Seqcluster was first converted into a FASTA file. The FASTA header was reformatted by including a sample‐specific three‐letter code, which is also a requirement for miRDeep2 analysis. For instance, ‘>A01_1_x446 A01’ represents sample C1033, whose first read was repeated 446 times.

The collapsed reads were mapped against the ovine reference genome (oar version 3.1) using bowtie (Langmead *et al.,* 2009). The Bowtie parameters were adjusted so that (i) the resulting alignments had no more than one mismatch (‐v 1); (ii) the alignments for a given read were suppressed if more than eight alignments existed for it (‐m 8); and (ii) the best‐aligned read was reported (‐‐strata, ‐‐best). The alignment outputs (in SAM format) were coordinate‐sorted and converted to BAM files. The sorted BAM files were converted to the miRDeep2 ARF format using the ‘bwa_sam_converter.pl’ script.

mirdeep2 version 2.0.0.5 (Friedländer *et al.,* 2012) was used to identify known ovine miRNAs and to predict conserved (known in other species) and novel ovine miRNAs. Before running the mirdeep2 pipeline, we merged both the collapsed FASTA files and the mapped ARF files. Furthermore, hairpin and mature sequences of all species were extracted from mirbase version 22 (Kozomara & Griffiths‐Jones, 2011, 2014). The extracted sequences were grouped into mature ovine sequences, ovine hairpin sequences and mature sequences for all species except sheep. The results from mirdeep2 were further processed to compile a list of all known and novel miRNAs. For novel and conserved miRNAs, we designated provisional IDs that included the genomic coordinates of the putative mature and star sequences.

From the list of miRNAs discovered by mirdeep2, those with a minimum count of 10 reads across all samples were considered for expression analysis. We used deseq2 (Love *et al.,* 2014) for differential expression analysis where the technical replicates of three samples (C107, C4271 and C312) were collapsed prior to running the DESeq command. We compared the expression level difference between breeds in each tissue separately. Differentially expressed miRNAs with an adjusted *p*‐value (*p*_adj_) of <0.1 were regarded as significant.

A total of 336.6 million reads was sequenced, of which approximately 42% contained adapters and/or low‐quality bases. After trimming, more than 92% of the reads (*n* = 311.3 million) were retained as high‐quality clean reads. On average, collapsing duplicate reads revealed 483 096 unique reads per sample, of which 54.4% of the unique sequences (collapsed reads) were mapped to the ovine reference genome. The detailed summary statistics for each sample are shown in Table S1. There were more collapsed reads and uniquely mapped reads for endometrial samples than CL samples despite the similar numbers of raw and clean reads in both tissues. After filtering out low count reads, a total of 599 miRNAs were included in the expression analysis. All of the miRNAs quantified in this study are presented in Table S2 and have been sent to be considered for adding to the next release of mirbase. The majority of the expressed miRNAs (*n* = 524) were shared by both tissues, with 43 and 32 miRNAs being unique to the CL and endometrium, respectively. Out of 599 miRNAs, 60 were conserved miRNAs in other species whereas 123 were known sheep miRNAs. Currently, 153 miRNAs are available in the mirbase database (Kozomara *et al.,* 2019). The database was updated to the current version (mirbase 22) from an earlier version (mirbase 21) after four years, and the overall number of miRNA sequences increased by over a third. However, the number of sheep miRNAs remained the same. Moreover, studies producing miRNA datasets have been scarce. As of April 2019, miRNA datasets from only three studies were available in the European Nucleotide Archive database, with accession nos PRJNA308631 (*n* = 3), PRJEB22101 (*n* = 37) and PRJNA414087 (*n* = 40); the PRJEB22101 dataset was from the first phase of this study (Pokharel *et al.,* 2018). In the current study, we quantified over threefold more sheep miRNAs (*n* = 599) than are available in mirbase. Therefore, these miRNAs will certainly improve the existing resources and will be valuable in future studies.

We did not perform differential gene expression analysis on the endometrial samples because of the sampling bias owing to embryos being of different ages. Two miRNAs, both upregulated in Finnsheep, were significantly differentially expressed between the pure breeds in the CL, whereas the other comparisons did not reveal any significantly differentially expressed miRNAs. Of these two significantly differentially expressed miRNAs, rno‐miR‐451‐5p is a conserved miRNA similar to that found in rat (*Rattus norvegicus*). The other, oar‐18_757_mt, is a novel miRNA expressed on chromosome 18. Chromosomal placement of the quantified miRNAs revealed a large cluster of miRNAs on chromosome 18 that we also observed in the ovaries (Fig. 1). The homologous region of this cluster has been identified and characterized in selected placental mammals such as human (chromosome 14), mouse (chromosome 12) and horse (chromosome 24) (Seitz *et al.,* 2004; Bentwich *et al.,* 2005; Dini *et al.,* 2018), but remain to be identified in other species. Using the region comparison tool in Ensembl, we were able to identify a similar cluster in elephant (chromosome 9), dog (chromosome 8), goat (chromosome 21), wild yak (scaffold CJH880931.1), pig (scaffold ScAEMK02000452.1MC), cow (chromosome 21) and others. With 46 miRNAs, the cluster in sheep is the largest, the closet being that in mouse, where the number of miRNAs is 44. This miRNA cluster is highly conserved among placental mammals and known to be regulated by imprinted regions (e.g. DLK1/DIO3) located approximately 200 kb upstream of the human (chromosome 14) and mouse (chromosome 12) miRNA clusters (Seitz *et al.,* 2004; Glazov *et al.,* 2008; Rocha *et al.,* 2008; Noguer‐Dance *et al.,* 2010). Therefore, we conclude that the sheep miRNA cluster in chromosome 18 is also maternally imprinted.

**Figure 1:**
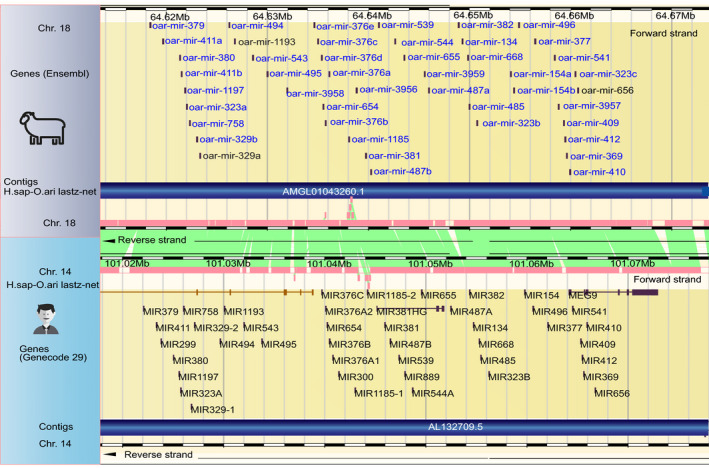
miRNA clusters in sheep (chromosome 18, top) and human (chromosome 14, bottom). Only three (marked in black ) out of 46 miRNAs in this cluster were not expressed in our data

## Conflict of interest

The authors declare that the research was conducted in the absence of any commercial or financial relationships that could be construed as potential conflicts of interest.

## Author contributions

J.K. M.H.L. and J.P. conceived and designed the project. K.P. analyzed the data and wrote the manuscript. All authors revised and approved the final manuscript.

## Funding

This study was funded by the Academy of Finland (decisions 250633, 250677 and 285017). This study is part of the ClimGen (‘Climate Genomics for Farm Animal Adaptation’) project funded by FACCE‐JPI ERA‐NET Plus on Climate Smart Agriculture. K.P. acknowledges financial support from the Niemi Foundation.

## Supporting information


**Table S1.** Summary of the miRNA‐Seq data

**Table S2.** List of miRNAs quantified in this study

## Data Availability

The raw FASTQ sequence data from this study have been deposited in the European Nucleotide Archive database under accession no. PRJEB32852. The secondary accession codes for each sample are included in the sample summary (Table S1) and the list of miRNAs quantified is available as Table S2 and has been submitted to Mirbase.
